# Mitochondrial DNA abnormalities provide mechanistic insight and predict reactive oxygen species-stimulating drug efficacy

**DOI:** 10.1186/s12885-021-08155-2

**Published:** 2021-04-17

**Authors:** Tarek Zaidieh, James R. Smith, Karen E. Ball, Qian An

**Affiliations:** 1 School of Pharmacy and Biomedical Sciences, Institute of Biological and Biomedical Sciences, University of Portsmouth, St Michael’s Building, White Swan Road, Portsmouth, PO1 2DT UK; 2 Institute of Life Science, Swansea University Medical School, Swansea, SA2 8PP UK

**Keywords:** Mitochondrial DNA, MtDNA variations, MtDNA copy number, Reactive oxygen species, Cisplatin, Dequalinium chloride hydrate, ROS-stimulating therapy, Cancer biomarker

## Abstract

**Background:**

Associations between mitochondrial genetic abnormalities (variations and copy number, i.e. mtDNAcn, change) and elevated ROS have been reported in cancer compared to normal cells. Since excessive levels of ROS can trigger apoptosis, treating cancer cells with ROS-stimulating agents may enhance their death. This study aimed to investigate the link between baseline ROS levels and mitochondrial genetic abnormalities, and how mtDNA abnormalities might be used to predict cancer cells’ response to ROS-stimulating therapy.

**Methods:**

Intracellular and mitochondrial specific-ROS levels were measured using the DCFDA and MitoSOX probes, respectively, in four cancer and one non-cancerous cell lines. Cells were treated with ROS-stimulating agents (cisplatin and dequalinium) and the IC50s were determined using the MTS assay. Sanger sequencing and qPCR were conducted to screen the complete mitochondrial genome for variations and to relatively quantify mtDNAcn, respectively. Non-synonymous variations were subjected to 3-dimensional (3D) protein structural mapping and analysis.

**Results:**

Our data revealed novel significant associations between the total number of variations in the mitochondrial respiratory chain (MRC) complex I and III genes, mtDNAcn, ROS levels, and ROS-associated drug response. Furthermore, functional variations in complexes I/III correlated significantly and positively with mtDNAcn, ROS levels and drug resistance, indicating they might mechanistically influence these parameters in cancer cells.

**Conclusions:**

Our findings suggest that mtDNAcn and complexes I/III functional variations have the potential to be efficient biomarkers to predict ROS-stimulating therapy efficacy in the future.

**Supplementary Information:**

The online version contains supplementary material available at 10.1186/s12885-021-08155-2.

## Background

Hallmarks of cancer include genome instability and alterations in metabolism, for the latter of which mitochondria are key regulators [1, 2]. Mitochondria are semiautonomous eukaryotic organelles that are considered as cellular energy powerhouses. They consist of thousands of proteins, mainly encoded by the nuclear genome, but also 13 encoded by their own genome [3, 4]. Mitochondrial DNA (mtDNA) is a small circular DNA of 16,569 bp, which codes for 2 rRNAs (12S and 16S), 22 tRNAs, and protein subunits for four of the five complexes of the oxidative phosphorylation system (OXPHOS), namely seven subunits of NADH dehydrogenase (complex I), one of cytochrome *c* reductase (complex III), three of cytochrome *c* oxidase (complex IV) and two of ATP synthase (complex V). The four subunits of respiratory enzyme complex II are totally encoded by nuclear DNA (nDNA), and are transcribed and translated in the cytoplasm and then transported into the mitochondria [5]. The displacement-loop region (D-loop) is a long noncoding region of mtDNA approximately 1.1 kbp long and contains regulatory sequences for replication and transcription. A number of proteins are implicated in the replication and transcription of mtDNA, and mitochondrial transcriptional factor A (TFAM) plays dual roles in these biological processes through its binding to D-loop [5]. The number of mitochondria that exist in a human cell ranges from hundreds to thousands, and each mitochondrion contains 2–10 mtDNA copies [6]. The synthesis and degradation of mtDNA is rapid and independent of the cell cycle so the dynamic equilibrium between the two processes determines the mitochondrial DNA copy number (mtDNAcn) (reviewed in Ref. [7]). MtDNAcn is strictly regulated during differentiation, and normal healthy cells with a high demand for ATP generation via OXPHOS have higher mtDNAcn than cells with a low ATP requirement [8].

The OXPHOS system is implicated in many cellular processes such as cell communication, differentiation and apoptosis. Consequently, OXPHOS dysfunction can lead to the development of several human diseases, including cancers [9]. For example, mutations in nuclear genes that encode the succinate dehydrogenase (SDH, MRC complex II) subunits can alter corresponding subunit structure and consequently affect MRC complex II activity, substrate levels, reactive oxygen species (ROS) production and cell redox state, which in turn activates oncogenic transcription factors to regulate expression of target genes and stimulate cancer cell proliferation [10, 11].

To date, mitochondrial dysfunction and various mtDNA alterations including point mutations, deletions and copy number changes have been observed in several human cancers such as breast, colon, stomach, kidney, thyroid, head and neck and ovarian cancers [12–14], and some linked with clinical parameters [15].

A total of 635 mutations have been reported in the D-loop region, including 510 point mutations, 56 deletions and 69 insertions. Additional mutations have been identified in the coding regions: 593 mutations in the complex I subunits, 343 mutations in the complex III, IV and V subunits, and 165 mutations in the tRNA and rRNA genes [16]. An increased mtDNAcn has been observed in endometrial [17], untreated head and neck [18], prostate [18], pancreatic [19] and colorectal [20] cancers compared to normal cells. On the contrary, a decrease of mtDNAcn has been associated with renal [21], thyroid [22], breast [23], previously treated head and neck [14], hepatic [24] and ovarian cancers compared to normal cells [25]. While mtDNA alterations such as mutations and changes in mtDNAcn are commonplace in cancers compared to normal cells, much more needs to be learnt about how they interrelate with each other and how they associate with underlying biological processes, including ROS production and ROS-mediated drug response.

Mitochondria are considered a primary intracellular site of ROS generation via OXPHOS during ATP generation [26]. ROS play important roles in cell signalling pathways such as growth, differentiation, metabolism and apoptotic cell death signalling (reviewed in Ref. [27]). ROS have been purported to have a double-edged-sword effect in cancer cells since low levels of ROS can play a critical role in promoting cell proliferation and invasion, whereas excessive levels of ROS can cause oxidative damage to intracellular bio-macromolecules and consequently induce cell death (reviewed in Ref. [28]). This makes cancer cells vulnerable to agents that further increase ROS levels, e.g. cisplatin (CDDP) that has been reported to cause excessive accumulation of ROS resulting in cytotoxic effect in various types of cancer such as ovarian cancer [29], breast cancer [30] and prostate cancer [31, 32]. The mitochondria-targeting compound, dequalinium chloride (DQA), has also been reported to demonstrate potent anticancer activity in vitro and in vivo in different malignancies due to the resulting damage to mtDNA and the inhibition of mitochondrial complex I rendering elevated ROS generation [33–35]. DQA belongs to a group of chemical agents knows as delocalised lipophilic cations (DLCs). Positively charged DLCs are drawn into mitochondria in response to the negative electric potential across the mitochondrial membrane. More importantly, the difference in mitochondrial transmembrane potential between normal and cancer cells can cause a ten-fold increase in DLC accumulation in cancer cells compared to their normal counterparts (reviewed in Ref. [36]), making the targeted mitochondrial accumulation of DLCs an attractive approach to cancer-specific therapy. Our previous study confirmed observations by other groups that DLCs such as DQA preferentially target mitochondria in cancer cells [37]. Furthermore, DQA may afford enhanced drug efficacy with reduced side effects due to its cancer-specific targeting property compared to non-cancer-specific / nucleus-targeting compounds such as CDDP, even though both DQA and CDDP induce ROS production [37].

The main objectives of our ongoing research are to prove the therapeutic potential of targeting mitochondria via DLCs in treating cancer and to identify effective biomarkers (such as ROS and specific mtDNA variations) that can predict drug response. Our previous study revealed a positive correlation between baseline intracellular ROS levels and drug response in human cancer cell lines. Therefore, baseline ROS levels have been proposed as a novel biomarker to predict drug sensitivity [37]. However, since measuring ROS in tissues is technically challenging, a more straightforward, e.g. genetic, biomarker to indicate the response to ROS-stimulating agents was sought.

Due to the complexity of the pathways for cancer development and resistance, which involves the functional cross-talk of mtDNA genes and their related nuclear genes, it is challenging to prove direct links between mtDNA variations and phenotype, one reason being the absence of routine laboratory methods for the genetic manipulation of mammalian mtDNA. However, one way of proving a link is to demonstrate a corresponding mitochondrial/cellular effect in cells/tissues that contain the mtDNA variation. However, more than one nuclear or mitochondrial variation may be present, potentially confounding the interpretation. Recently, we have demonstrated that 3D structural analysis of mitochondrial proteins in silico can complement the former approach revealing detailed mechanistic insights into the functional role of variations within mtDNA encoded-OXPHOS genes [10].

Consequently, the present study aimed to explore the interrelationship between various mitochondrial genetic abnormalities (mtDNA variations and mtDNAcn change), baseline intracellular ROS levels and sensitivity to ROS-stimulating drugs such as CDDP and DQA within the same panel of cell lines employed in our previous study [37]. Here we show that mtDNAcn and the total number of non-synonymous variations within the mitochondrial genome have a strong correlation with both baseline ROS level and drug resistance. 3D structural modelling of these reveals a subset of variations that could be responsible for the observed mtDNA genotype-mitochondrial/cellular phenotype links. While repeating these experiments with the different mtDNAs on a standardised nuclear genetic background is warranted, we propose that this is a useful first step in the characterisation of the cancer cell lines, and reveals a subset of variations that have the potential to be used to stratify patient into subgroups that are most likely to benefit from ROS-stimulating agents in the future.

## Methods

### Cell culture, drug treatment, and functional assays

Four cancerous (Ishikawa/endometrium, MDA-MB-231/breast, Caco-2/colon, PC-3/prostate) and one non-cancerous (PNT-2/prostate) cell lines, two ROS-stimulating drugs (cisplatin (CDDP) and dequalinium chloride hydrate (DQA)), the MTS assay to determine cell viability and IC50s, and the DCFDA and MitoSOX probes detected with a microplate reader to measure baseline intracellular ROS and mitochondrial superoxide levels of cells, respectively, were employed in the study. Full methodological details of how these procedures were undertaken have been previously described [37]. All cell lines employed in the study were originally purchased from either the European Collection of Authenticated Cell Cultures/ECACC (Ishikawa, MDA-MB-231, Caco-2, PNT-2) or the American Type Culture Collection/ATCC (PC-3).

### DNA extraction and measurement of mtDNA copy number by SYBR Green real-time PCR

DNA extraction and mtDNA copy number measurement were also carried out as previously described [37].

### Mitochondrial DNA sequencing and database mining

To assess the mtDNA variations of the cell lines in this study seventeen primer pairs were designed using Primer-BLAST (https://www.ncbi.nlm.nih.gov/tools/primer-blast/index.cgi) to amplify the entire mitochondrial genome in overlapping fragments of ~ 1.1 kb (Primer sequences, location, and expected PCR amplicon sizes are listed in Table S5).

All PCR amplifications were performed in a 50-μl reaction containing 1 × PCR buffer, 1.5 mM MgCl_2_, 0.2 mM dNTPs, 0.2 μM of each forward and reverse primer, 1.25 U GoTaq® G2 Hot Start Polymerase (Promega, Southampton, UK) and 200 ng of DNA. The amplification procedure entailed 95 °C for 15 min, followed by 35 cycles of 95 °C for 40 s, 60 °C for 40 s and 72 °C for 2 min with a final elongation step for 10 min at 72 °C. PCR products were visualised by gel electrophoresis using 1.2% agarose gel at 125 V for 50 min.

Sanger sequencing, with a read length of 900-1000 bp, of the PCR products was performed using forward and reverse primers by Eurofins Genomics (Ebersberg, Germany), yielding a 2-fold coverage and whole mitochondrial genomes in each case. Variations in the mitochondrial genome were identified by comparing the obtained sequences with the NCBI Human Mitochondrial Reference Sequence (NC_012920.1; https://www.ncbi.nlm.nih.gov/nuccore/251831106/) using the Nucleotide BLAST software (https://blast.ncbi.nlm.nih.gov/Blast.cgi). Identified mtDNA variations were annotated and their disease association was analysed using online tools and databases including MitoWheel (http://mitowheel.org/mitowheel.htmL), MitoMAP (https://www.mitomap.org/MITOMAP) and Human Mitochondrial DataBase (HmtDB; https://www.hmtdb.uniba.it/).

The HmtDB, mtDB (http://www.mtdb.igp.uu.se/) and MitoMAP were also used to determine the frequencies of functional mtDNA variations and 310InsC in the D-loop region in the healthy tissues.

### In silico three-dimensional protein structure mapping and analysis of variations

A three-dimensional (3D) protein structure mapping and analysis approach (developed and validated by Lloyd and McGeehan) [38–40] was used to predict the functional impact of all non-synonymous identified in mtDNA protein coding regions.

Briefly, the human mtDNA-encoded protein sequences were used to identify the most homologous OXPHOS complexes I, III, IV and V available from the RCSB PDB (Research Collaboratory for Structural Bioinformatics Protein Data Bank; http://www.rcsb.org/). Then, the best quality, latest and most similar complex structures were choosen; complex I – [PDB:5XTD] (*Homo sapiens*) [41]; complex III – [PDB:5XTE] (*Homo sapiens*) [41]; complex IV [PDB:5Z62] (*Homo sapiens*) [42] and complex V [PDB:5ARA] (*Bos taurus*) [43].

The atomic co-ordinates of each complex were downloaded from the PDB and opened using PyMol (the PyMOL Molecular Graphics System/Version 1.8; distributed by Schrödinger, LLC, NY, USA). All identified non-synonymous variation sites were mapped to their amino acid locations on the protein structures using PyMol. COOT software (Crystallographic Object-Oriented Toolkit) was also used to create PDB files containing all non-synonymous variations identified. PDB files including the variations (created by COOT) were also opened with PyMol, and compared with the wild-type structures. Protein structures were displayed in a cartoon style, and each variation was identified and displayed in the stick and sphere format.

Detailed analysis of the potential effect of each variation on protein structure was performed by examining the type of amino acid change, as well as its location within the protein. Factors taken into consideration included: proximity to important catalytic regions, such as active sites, binding pockets and subunit interfaces.

### Statistical analysis

All data are expressed as mean ± SEM obtained from a minimum of three independent experiments. Statistical analyses and graphical representations were produced using the GraphPad Prism 8.0 program (Graphpad Software, CA, USA). More specific descriptions of the statistical tests used to analyse individual data sets are provided in the associated figure legends. Differences between groups were considered statistically significant based on the following criteria: *p < 0.05, **p < 0.01, ***p < 0.001 and ****p < 0.0001.

Potential linear correlation between two variables was assessed by the Pearson correlation coefficient method using SPSS (2015) software. Pearson’s ***r*** has a value between + 1 and − 1, where + 1 indicates a total positive linear correlation, 0 indicates a non-linear correlation, and − 1 indicates a total negative linear correlation.

## Results

### Baseline intracellular ROS, mitochondrial superoxide and drug response levels

**Fig. 1:**
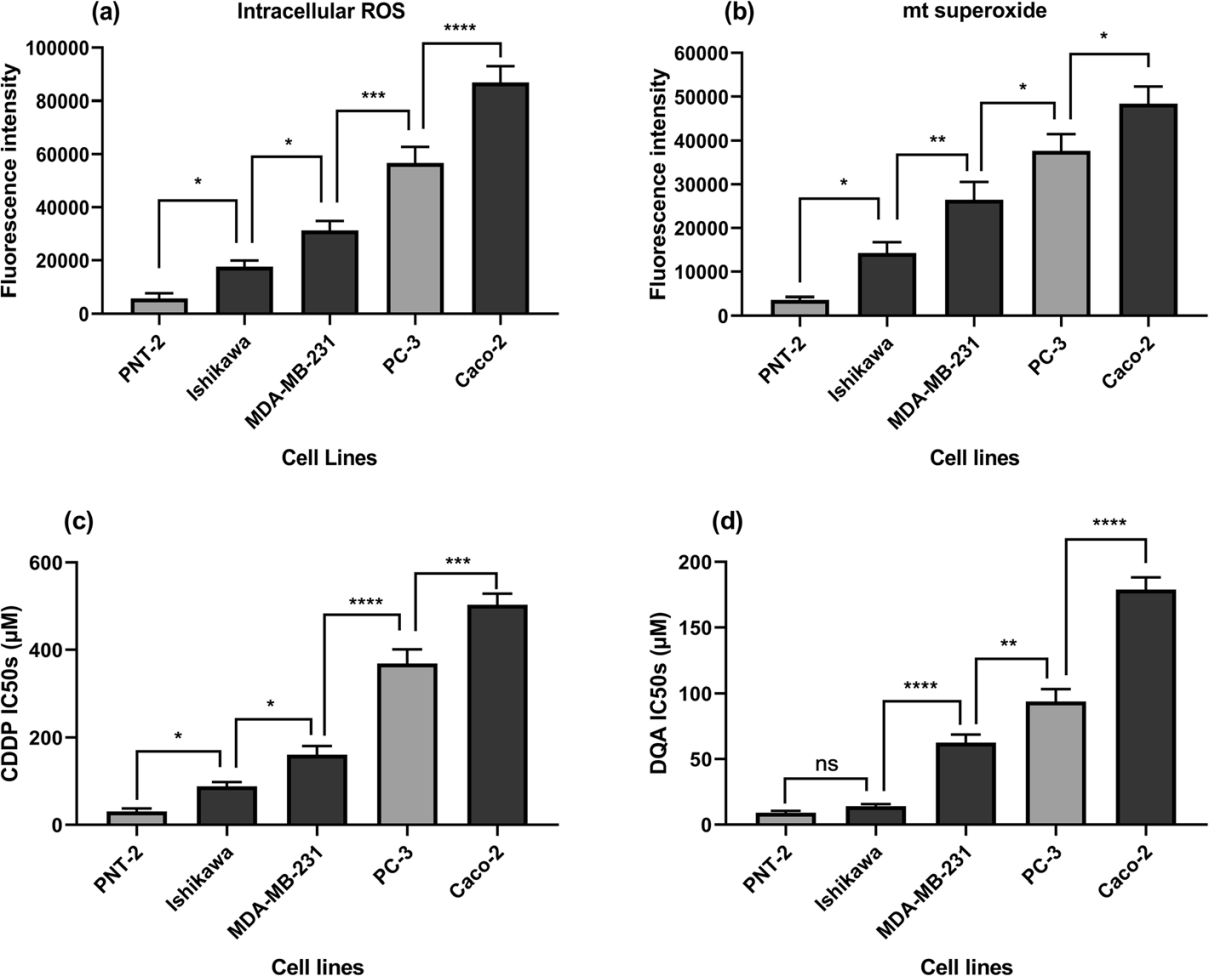
Intracellular ROS (**a**) and mitochondrial superoxide (**b**) levels of the PNT-2, Ishikawa, MDA-MB-231, PC-3 and Caco-2 cell lines are represented by the fluorescence intensity of DCFDA and MitoSOX respectively. Comparison of the CDDP IC50s and the DQA IC50s amongst the 5 cell lines. The columns represent the CDDP IC50s (**c**) and the DQA IC50s (**d**) in the all cell lines. Data are mean ± SEM (*N* = 3 separate experiments); *p* values were calculated using one-way ANOVA with Tukey multiple comparison post-hoc analysis; ns, not significant, *p < 0.05, **p < 0.01, ***p < 0.001 and ****p < 0.0001

Intracellular ROS and mitochondrial superoxide levels were measured using two fluorescence-labelled probes, DCFDA and MitoSOX, respectively. Results from those assays indicated that the cancer cell lines had increased baseline intracellular ROS and mitochondrial superoxide levels compared to the non-cancerous cells (PNT-2) (Fig. 1a & b). Amongst the cancer cell lines, Caco-2 had the highest baseline intracellular ROS and mitochondrial superoxide levels whereas Ishikawa had the lowest. Drug sensitivity levels towards CDDP and DQA were evaluated for all cell lines by calculating their IC50s. Data in Fig. 1c & d show that the non-cancerous PNT-2 cells were more sensitive to both drugs compared to the cancer cells with Caco-2 being the most resistant whereas Ishikawa the most sensitive amongst the cancer cells, although in terms of the DQA treatment, PNT-2 was not significantly more sensitive compared to Ishikawa (Fig. 1a).

### Mitochondrial genetic profiles

**Fig. 2:**
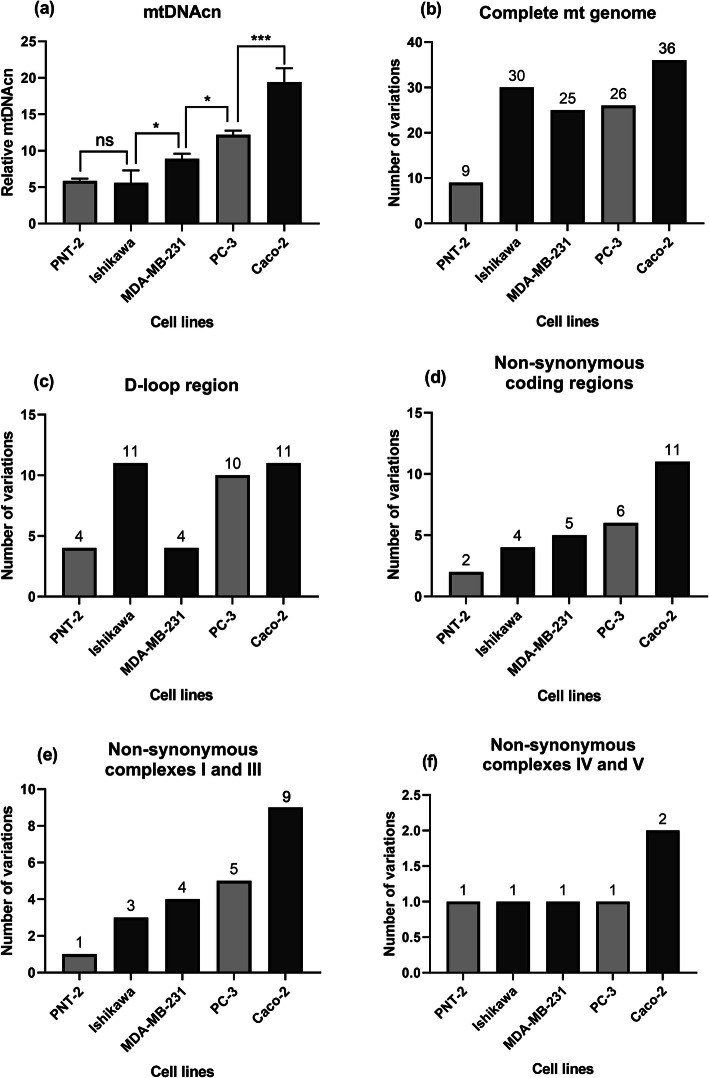
Mitochondrial genetic profiles for PNT-2, Ishikawa, MDA-MB-231, PC-3 and Caco-2. **a** Relative content of mtDNA in the all cell lines normalised against the house keeping gene (*β*-actin). The columns represent the relative mtDNAcn of the cell lines. Data are mean ± SEM (*N* = 3 separate experiments); *p* values were calculated using one-way ANOVA with Tukey multiple comparison post-hoc analysis; ns, not significant, *p < 0.05 and ***p < 0.001. **b** Numbers of the total variations observed in the cell lines. **c** Numbers of variations identified in the D-loop region in the cell lines. **d** Numbers of non-synonymous variations observed in the whole mtDNA protein coding regions. **e** Numbers of non-synonymous variations observed in mtDNA coding regions for the complex I and III subunits. **f** Numbers of non-synonymous variations observed in mtDNA coding regions for the complex IV and V subunits

Relative quantification of mtDNAcn was performed using the SYBR Green PCR technique. The results showed that cancer cell lines also had increased mtDNAcn compared to the non-cancerous cells. Amongst the cancer cell lines, Caco-2 had the highest baseline mtDNAcn whereas Ishikawa had the lowest (Fig. 2a). The complete mitochondrial genome of each cell line was PCR amplified via 17 overlapping amplicons and the PCR products were confirmed by agarose gel electrophoresis (a representative gel image is shown in Figure S1). Sequencing data revealed that the total number of mtDNA variations in each cancer cell line was comparable (30, 25, 26 and 36 variations in Ishikawa, MDA-MB-231, PC-3 and Caco-2, respectively). However, these numbers were significantly higher than that in the non-cancerous cell line (PNT-2), in which only 9 variations were identified (Fig. 2b).

Variations within the D-loop region were identified and Ishikawa, PC-3 and Caco-2 showed greater numbers of variations in this region compared to PNT-2 and MDA-MB-231 (Fig. 2c). The sequencing data revealed that the D-loop region was a hotspot for variations identified in the present study. Numerous common variations amongst the cancer cell lines were observed in the D-loop region (summarised in Table S1). Our results indicated that A73G was detected in Ishikawa, PC-3 and Caco-2; T195C was identified only in MDA-MB-231 and PC-3; A263G was a common variation in all the cancer cell lines; 310InsC was observed only in Ishikawa, PC-3 and MDA-MB-231; T16172C was detected in Ishikawa and PC-3; C16261T occurred in Ishikawa and Caco-2. None of the common variations seen in the cancer cells was detected in the non-cancerous cell line (PNT-2). On the other hand, each cell line harboured a number of unique variations in the D-loop region (listed in Table S2). 513InsCA, G16319A, T16325C and T16519C were found only in the PNT-2 cells; C150T, C338T, C16223T, C16257A, T16304C and A16497G were found only in Ishikawa; A153G was found only in MDA-MB-231; T195C, C16192T, C16256T, C16270T, C16320T and A16399G were found only in PC-3; G47A, G185A, G228A, C295T, C462T, T489C, C16069T and T16126C were found only in Caco-2.

Progressive increases in the number of non-synonymous point variations in the protein coding regions (2, 4, 5, 6, 11) were observed in PNT-2, Ishikawa, MDA-MB-231, PC-3 and Caco-2, respectively (Fig. 2d). The same trend was observed for non-synonymous point variations in complexes I and III (1, 3, 4, 5 and 9 variations) (Fig. 2e), but not complexes IV and V (Fig. 2f). Numerous common non-synonymous variations among the cell lines were observed within the mitochondrial genome (Table S1). Our results indicated that the A8860G variation was detected in all the cell lines whereas C14766T was detected only in the cancer cells. G13708A was identified only in the PC-3 and Caco-2 cells, whereas A15326G was a common variation in the PNT-2, Ishikawa, MDA-MB-231 and Caco-2 cells. On the contrary, greater numbers of unique non-synonymous variations, mainly in complexes I and III, were observed in the cancer cells (Table S2). The T3394C point variation was found only in the Ishikawa cells; C12084T and A13966G were found only in the MDA-MB-231 cells; T11120C, C13802T and A14793G were found only in the PC-3 cells. Caco-2 had the highest number of unique variations including T4216C, G7977C, A10398G, A13681G, T14798C, A14927G and C15452A. No unique variations within complexes I and III were found in the non-cancerous cells (PNT-2).

### Positive correlations between cellular and genetic parameters

In order to investigate how mitochondrial genetic abnormalities might affect the baseline ROS level and drug response in the cancer cells, linear regression was employed to model the relationship between the redox statuses and CDDP/DQA IC50s. The relationship between these cellular parameters and each of the following genetic parameters: mtDNAcn, total variations, D-loop variations, non-synonymous variations, and complexes I/III non-synonymous variations, was also investigated.

**Fig. 3:**
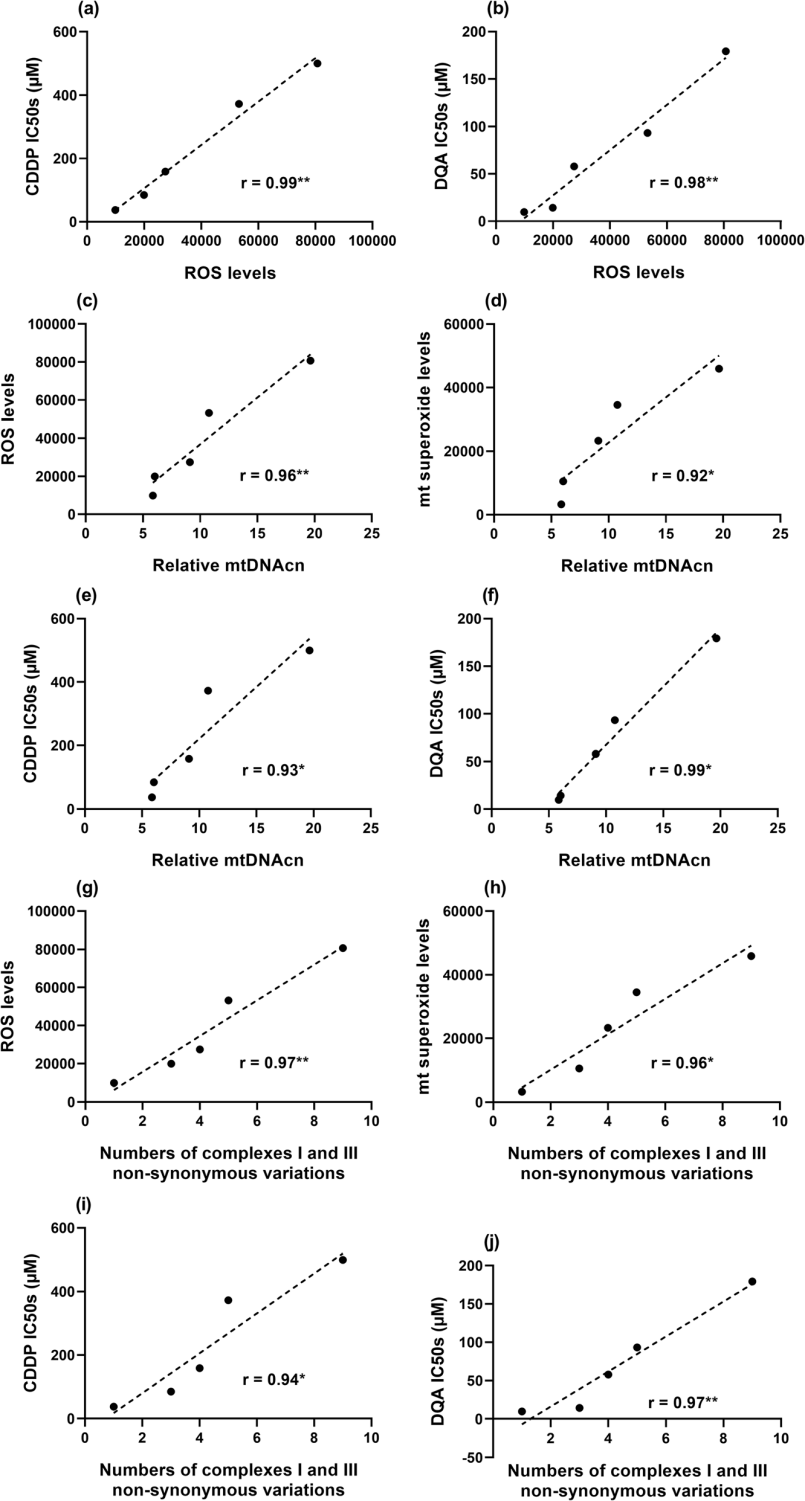
Positive correlation between the baseline intracellular ROS level and drug resistance against CDDP (**a**) and DQA (**b**). Positive correlations between the relative content of mtDNA and the baseline intracellular ROS level (**c**), mitochondrial superoxide level (**d**), CDDP IC50s (**e**), DQA IC50s (**f**). Positive correlations between the numbers of non-synonymous variations in the complexes I & III coding regions and baseline ROS levels (**g**), mitochondrial superoxide levels (**h**), CDDP IC50s (**i**), DQA IC50s (**j**). Data points representing the cell lines are in the sequence of PNT-2, Ishikawa, MDA-MB-231, PC-3 and Caco-2. r and *p* and values were calculated using the Pearson correlation coefficient method; *p < 0.05 and **p < 0.01

Baseline intracellular ROS levels were positively correlated with the resistance levels to both drugs in the cell lines (Fig. 3a & b), confirming our previous observations [37]. Positive correlations between mtDNAcn and intracellular ROS, mitochondrial superoxide (Fig. 3c & d), and resistance levels to both drugs (Fig. 3e & f) in the cell lines were also observed between the cell lines.

Data in Fig. 3g & h show that numbers of variations in the complex I and III coding regions were positively correlated with the overall ROS and mitochondrial superoxide levels. Those non-synonymous variation numbers were also positively correlated with the IC50s of both CDDP and DQA (Fig. 3i & j). Similarly, numbers of non-synonymous variations identified in the coding regions for all the mitochondrial OXPHOS subunits were also positively correlated with intracellular ROS, mitochondrial superoxide and CDDP/DQA IC50s (data not shown). Neither the D-loop variations or total number of variations correlated with ROS levels, drug sensitivity or mtDNAcn (Fig. S2 & 3). These findings indicate that the following parameters, mtDNAcn, total non-synonymous variations and non-synonymous variations in complexes I/III, could serve as biomarkers for ROS levels and ROS-stimulating drug resistance.

### Eight functional variations identified in complexes I and III

There were 17 non-synonymous and 20 synonymous variations identified in complexes I, III, IV and V. Amongst the complex I variations, 9 were non-synonymous substitutions, of which 2 were located in MT-ND1, 1 in MT-ND3, 2 in MT-ND4 and 4 in MT-ND5. As to the complex III variations, 6 MT-CYB substitutions were identified in total. For the remaining 2 non-synonymous variations, 1 substitution was found in complex IV affecting MT-COII and 1 substitution was identified in the MT-ATP6 subunit of complex V.

The 3D structural analysis revealed that of the 17 non-synonymous variations identified within the mtDNA protein-coding regions, 8 were predicted to be functional, all in complex I (A10398G (T114A), T11120C (F121L), C12084T (S442F), A13681G (T449A), G13708A (A458T), C13802T (T489M), A13966G (T544A)) and complex III (T14798C (F18L)). No functional variations were predicted in complexes IV and V. None of the variations predicted to be functional were found in the non-cancerous PNT-2 cells.

All the non-synonymous variations and their structural modelling predictions are summarised in Table 1.

**Table 1:** Summary of the non-synonymous variations and the 3D structural modelling results

Variations	Locus	Complex	Amino Acid change	Sequence	Location	Interaction	Prediction	Occurrences of functional variations	Ref.
T3394C	MT-ND1	I	Y30H	Conserved	Surface	None	Non-functional		
T4216C	MT-ND1	I	Y304H	Non-conserved	Surface	None	Non-functional		[39]
A10398G	MT-ND3	I	T114A	Non-conserved	Surface	MT-ND3 & MT-ND1	Functional	Caco-2	
T11120C	MT-ND4	I	F121L	Conserved	Surface	MT-ND4 intra-subunit	Functional	PC-3	
C12084T	MT-ND4	I	S442F	Conserved	Core	MT-ND4 intra-subunit	Functional	MDA-MB-231	
A13681G	MT-ND5	I	T449A	Conserved	Surface	MT-ND5 Intra-subunit	Functional	Caco-2	
G13708A	MT-ND5	I	A458T	Conserved	Core	MT-ND5 Intra-subunit	Functional	Caco-2 & PC-3	
C13802T	MT-ND5	I	T489M	Conserved	Surface	MT-ND5 Intra-subunit	Functional	PC-3	
A13966G	MT-ND5	I	T544A	Conserved	Surface	MT-ND5 Intra-subunit	Functional	MDA-MB-231	
C14766T	MT-CYB	III	T7I	Conserved	Surface	None	Non-functional		[39, 40]
A14793G	MT-CYB	III	H16R	Conserved	Surface	None	Non-functional		[39, 40]
T14798C	MT-CYB	III	F18L	Conserved	Surface	Substrate binding site	Functional	Caco-2	[10, 40]
A14927G	MT-CYB	III	T61A	Conserved	Surface	None	Non-functional		
A15326G	MT-CYB	III	T194A	Conserved	Surface	None	Non-functional		[39, 40]
C15452A	MT-CYB	III	L236I	Conserved	Surface	None	Non-functional		[39, 40]
G7977C	MT-CO2	IV	G131A	Conserved	Surface	None	Non-functional		
A8860G	MT-ATP6	V	T112A	Non-conserved	Surface	None	Non-functional		

**Fig. 4:**
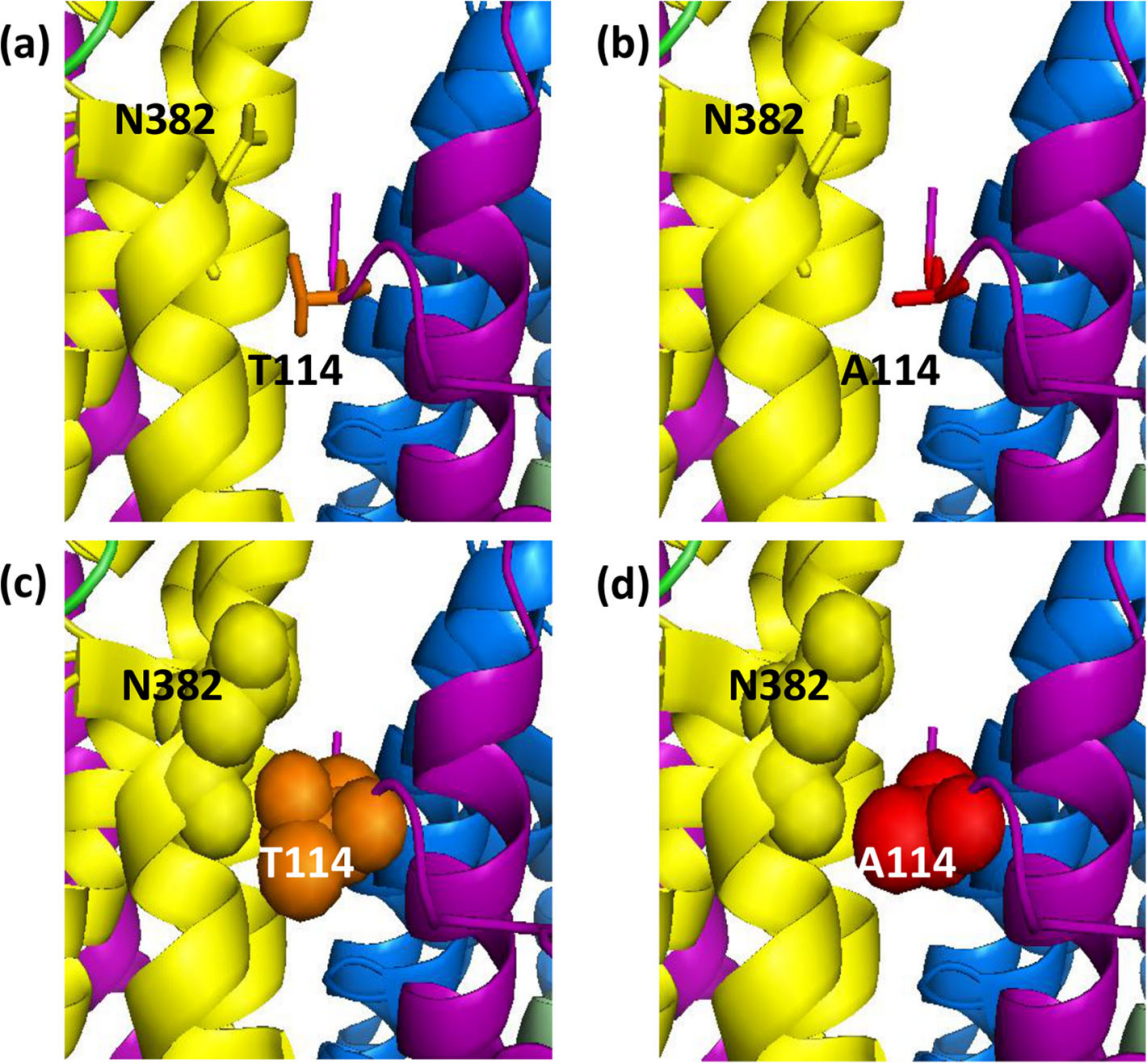
Detailed view of the complex I variation A10398G (T114A). T114 is located at the surface of complex I within the mitochondrial DNA encoded MT-ND3 subunit. MT-ND3 is shown in purple and MT-ND1, an adjacent subunit, in yellow. The *wild type* T114 is shown in orange as sticks and spheres (**a** & **c**, respectively) and the mutant A114 is shown in red (**b** & **d**). Alanine is non-polar and smaller than threonine in size meaning the change is likely to result in the loss of hydrogen bonds (dotted line) with the N382 residue of MT-ND1, and therefore affect the association of the two subunits and consequently the stability of complex I

**Fig. 5:**
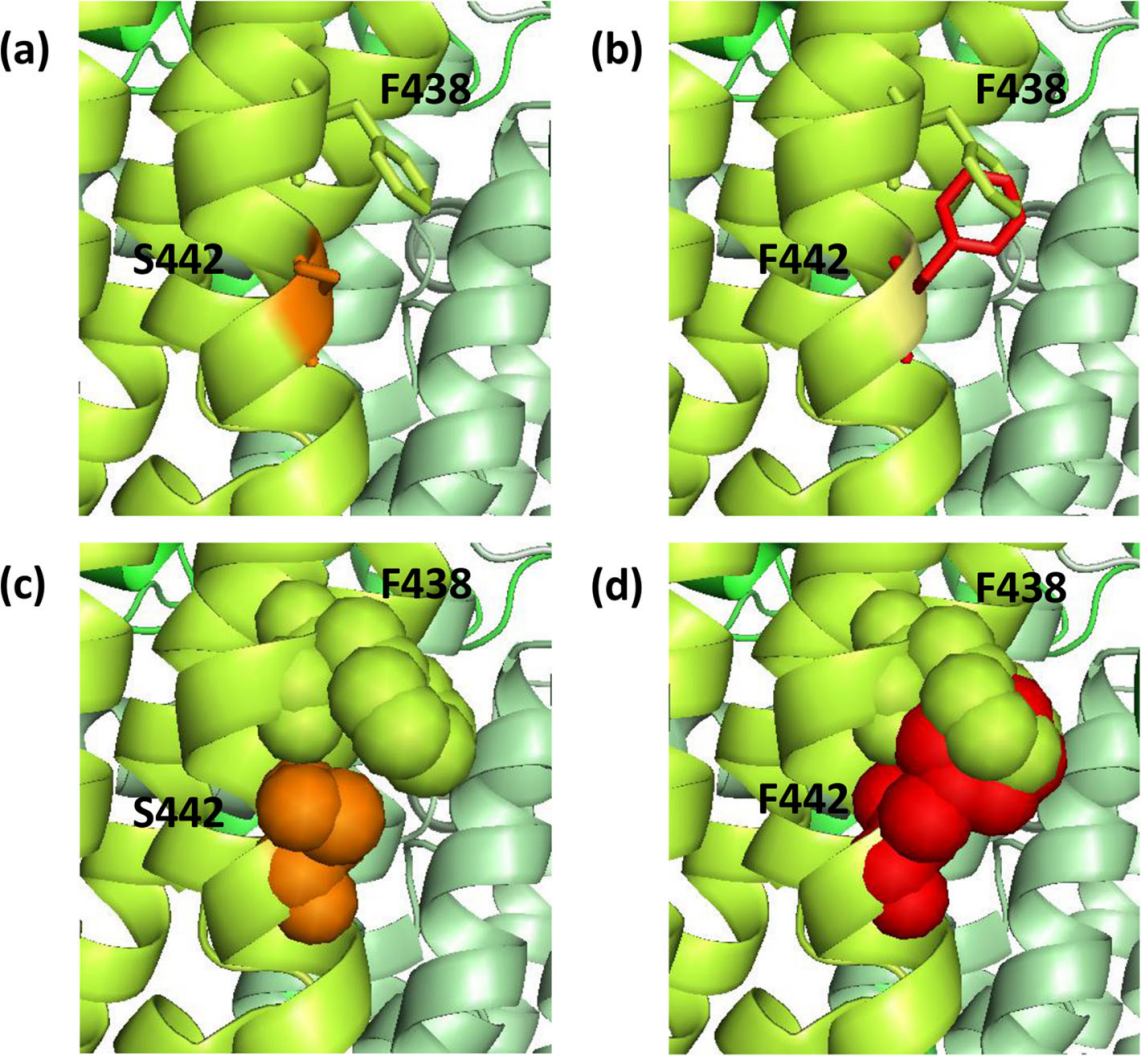
Detailed view of the complex I variation C12084T (S442F). S442 is located at the core of complex I within the mitochondrial DNA encoded MT-ND4 subunit. MT-ND4 is shown in bright green. The *wild type* S442 is shown in orange sticks and spheres (**a** & **c**, respectively) and the mutant F442 is shown in red (**b** & **d**). Phenylalanine is bigger than serine due to its aromatic side chain and is likely to disrupt and repel the hydrophobic F438 residue within MT-ND4, which is predicted to affect the stability of complex I

**Fig. 6:**
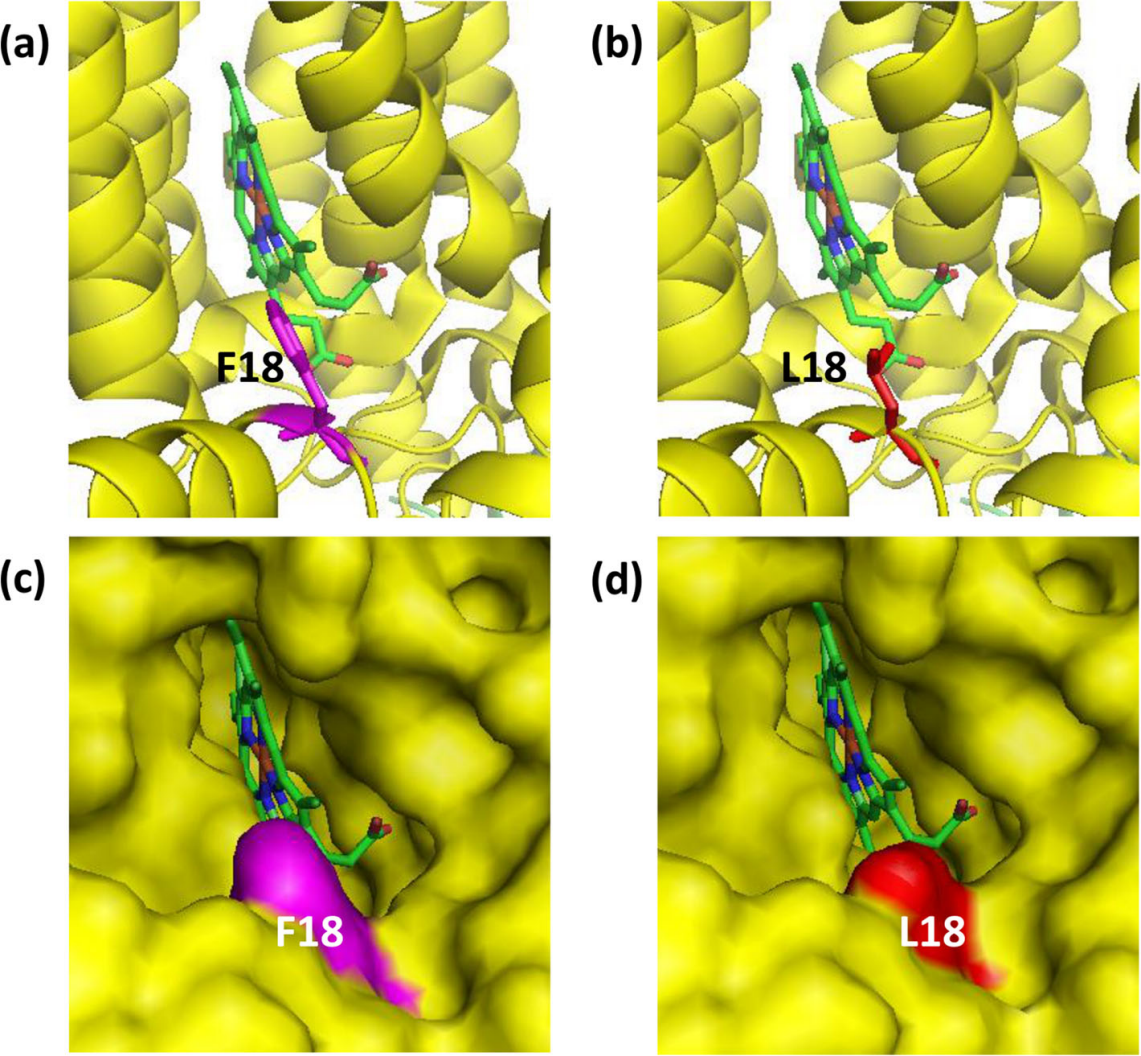
Detailed view of the complex III variation T14798C (F18L). F18 is located at the entrance of the ubiquinone-binding site (Q_i_ site) within the mitochondrial DNA encoded cytochrome *b* subunit (MT-CYB). MT-CYB is shown in yellow and ubiquinone in green. The *wild type* F18 is shown in purple sticks and spheres (**a** & **c**, respectively) and the mutant L18 is shown in red (**b** & **d**). Phenylalanine is a much larger residue than leucine, suggesting that this change is likely to affect the shape and structure of the binding site, the on/off rate of ubiquinone, and consequently alter the activity of complex III

Figure 4, 5 and 6 depict the structural consequences of the complex I and III variations predicted to be functional. The A10398G mutation, presents in Caco-2 with the highest baseline ROS and drug IC50, could cause the disruption of complex I assembly and stability by affecting the association of MT-ND1 and MT-ND3. The replacement of T114 by A114 removes the polar sidechain property of threonine, which is likely to affect the formation of hydrogen bonds with the MT-ND1 residue (N382) and weaken the interaction between the two subunits, and consequently affect the assembly of the two subunits and the stability of complex I (Fig. 4). Similarly, the A13681G, C13802T and A13966G variations, present in Caco-2, PC-3 and MDA-MB-231 respectively, cause the disruption of MT-ND5 subunit stability due to the loss of the hydrogen bonds formed between the residues within the subunit. Consequently, these variations are all predicted to weaken the interaction within the subunit, hence affect the stability of complex I.

The C12084T mutation, presents in MD-MB-231, results in the disruption of MT-ND4 stability. This variation replaces serine with phenylalanine (a larger amino acid) at position 442 (S442F) which is unlikely to be accommodated within the structure. Apart from being of a larger size, this phenylalanine at position 442 may disrupt and repel another phenylalanine nearby within the MT-ND4 subunit (i.e. F438) and consequently affect the stability of complex I (Fig. 5). Similarly, the T11120C variation in PC-3 and G13708A variation in PC-3 and Caco-2 cause the disruption of the MT-ND4 and MT-ND5 subunits respectively due to the lack of accommodation capacity for the newly formed residues. These intra-subunit disruptions could interfere with the stabilisation of the MT-ND4 and MT-ND5 subunits and consequently affect the stability of complex I.

Complex III is at the centre of the OXPHOS system and the MT-CYB subunit lies at the centre of dimeric complex III containing two haems and two inhibitor binding sites, the Qo and Qi sites. T14798C in Caco-2 that results in the amino acid change F18L is located at the ubiquinone-binding site (i.e. the Q_i_ site) of the MT-CYB subunit. Phenylalanine (F) is one of the residues that form the entrance of the Q_i_ site of complex III [44]. Both phenylalanine (F) and leucine (L) are hydrophobic. However, they differ in their side chains. Leucine does not have an aromatic side chain, which makes it smaller than phenylalanine. Therefore, this substitution is likely to alter the shape and size of the Q_i_ site because it results in a larger cavity at the entrance to the site (Fig. 6). Such a change may interfere with the binding and dissociation of ubiquinone, the Qi site ligand, and consequently the electron transfer from heme *b*_H_.

On the contrary, nine variations were predicted to be non-functional. These were: T3394C (Y30H) and T4216C (Y304H) in complex I; C14766T (T7I), A14793G (H16R), A14927G (T61A), A15326G (T194A) and C15452A (L236I) in complex III; G7977C (G131A) in complex IV; A8860G (T112A) in complex V. The structural analysis revealed that all those residues were distal from any known electron or proton route or major catalytic sites, and did not have any direct interaction with amino acids from neighbouring mitochondrial or nuclear subunits. There also appeared to be sufficient room to accommodate the amino acid change in each case.

**Fig. 7:**
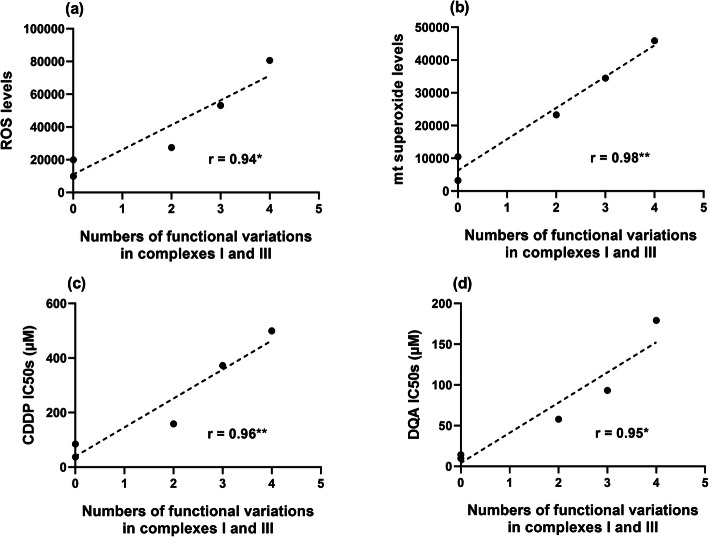
Positive correlations between the numbers of functional variations predicted in the complex I & III coding regions and baseline ROS levels (**a**), mitochondrial superoxide levels (**b**), CDDP IC50s (**c**) and DQA IC50s (**d**). Data points representing the cell lines are in the sequence of PNT-2, Ishikawa, MDA-MB-231, PC-3 and Caco-2. r and p and values were calculated using the Pearson correlation coefficient method; *p < 0.05 and **p < 0.01

The combined effect of individual non-synonymous variations predicted to be functional in our study by 3D structural modelling is expected to be magnified if more than one is present in a cell/cell line, and so it is noteworthy that there was a positive correlation between the number of non-synonymous variations in complexes I and III predicted to be functional with the overall ROS and mitochondrial superoxide levels, and CDDP/DQA IC50s (Fig. 7). This finding suggests that an increasing number of non-synonymous variations predicted to have an effect on the structure and function of complexes I and III could provide a mechanistic link to the other effects observed, i.e. associated increase in ROS, drug resistance and mtDNAcn.

This can also be illustrated by comparing the cancerous PC-3 and non-cancerous PNT-2 cell lines, both of which originated from prostate tissue. Clearly, all the parameters, including baseline intracellular ROS and mitochondrial superoxide levels, CDDP and DQA resistance, mtDNA copy number, total number of variations, total number of D-loop variations, total number of non-synonymous point mutations in protein coding regions and in complexes I and III, are elevated in PC-3 compared to PNT-2. Furthermore, it is worth noticing that the PC-3 cells contains three variations predicted to be functional (C13802T, G13708A, and T11120C), whereas the PNT-2 cells do not contain any.

### Varying frequency in healthy tissues and heritability of functional variations

Among the subset of 8 variations classified as functional, only three: A10398G, G13708A and T14798C occur frequently in healthy tissues, i.e. more than 500 in 30,000 (1.6%) of complete mitochondrial genomes in at least 2 out 3 of the consulted databases (Table S3). On the other hand, 5 of the functional variations: A13966G, T11120C, C12084T, A13681G and C13802T as well as the D-loop variation 310InsC occur rarely in healthy tissues, i.e. less than 1.6% of the complete mitochondrial genomes in at least 2 out of 3 of the databases consulted (Table S3). A MitoMAP search of the subset of the 8 functional variations and 310IncC revealed that 5 have not been previously reported as either somatic or inherited variations, i.e. they are of unknown heritability (T11120C, C12084T, A13681G, C13802T, and 310InsC); three have been reported as somatic or inherited (A10398G, G13708A and T14798C), and one has been reported as inherited (A13966G) (Table S3). While our results indicate that variations occurring at low frequencies in healthy tissues are more likely to be previously unreported variations of potential functional importance, the possibility that previously reported somatic or inherited variations occurring at high frequencies in healthy tissues could also play a role in increased baseline ROS level, drug resistance and mtDNAcn cannot be ruled out.

## Discussion

It has become broadly acknowledged that the redox status of cancer cells could be manipulated to achieve cancer-specific killing since high levels of intracellular ROS activate death signal pathways (reviewed in Ref. [27]). Given that mitochondria are a primary intracellular site of ROS production via OXPHOS during ATP generation [26], it is not surprising that mitochondria have attracted increasing research interest as a promising target for anticancer therapy. It has been reported that mtDNA mutations could influence intracellular ROS levels [45, 46]. In particular, since complexes I and III are the main ROS generation sites, variations in their mtDNA genes could have a significant impact on the overall intracellular ROS level (reviewed in Ref. [47]). However, how specific mtDNA variations influence the efficacy of mitochondria-targeting/ROS-stimulating therapy remains unclear. Our previous data have shown that elevated baseline intracellular ROS levels among different cancer cell lines correlate positively with increased resistance towards cisplatin and dequalinium, both being ROS-stimulating drugs [37]. We hypothesised that certain mitochondrial genetic abnormalities, including variations and copy number (mtDNAcn) changes, could influence intracellular ROS levels, and therefore be utilised as biomarkers to predict cancer cells’ response to ROS-stimulating agents. The present study characterised the interrelationship between mitochondrial genetic parameters (variation and mtDNAcn), ROS levels and ROS-mediated drug response with the aim of generating future novel biomarkers that have a significant association with drug response of cancer cells. Such biomarkers have the potential to be integrated into future clinical practice in order to facilitate personalised medicine.

As a first step to investigating the possible link between mitochondrial genetic abnormalities and the cells’ response to ROS-stimulating agents, a range of molecular, biochemical and bioinformatics methods were employed in the present study to analyse ROS levels, cytotoxicity of cisplatin and dequalinium, as well as mtDNA variations and relative copy numbers in 4 cancer and 1 non-cancerous cell lines (two of the cell lines, cancerous PC-3 and non-cancerous PNT-2, originated from the same tissue type, i.e. prostate).

We found that cancer cells generally carry more variations and a higher mtDNAcn in their mitochondrial genome than non-cancerous cells and have higher baseline ROS levels. This is consistent with the observations of others [47–49] and likely reflects the cancer cells’ adaption to oxidative stress via enhanced DNA repair mechanisms, upregulated anti-apoptotic pathways and consequently increased drug resistance/cell survival (reviewed in Ref. [28]).

We also confirmed our previous observation that increasing baseline ROS level correlates positively with increasing resistance to the ROS-stimulating agents in cancer cell lines. We further demonstrated that increased ROS and drug resistance levels also correlate positively with greater mtDNAcn, higher number of non-synonymous complex I and III variations, and higher number of complex I and III functional variations predicted by the 3D structural modelling (i.e. A10398G, T11120C, C12084T, A13681G, G13708A, C13802T, A13966G and T14798C) (Table S4). The positive correlation between the number of predicted complex I/III functional variations and ROS levels appears more significant for mitochondrial ROS than for overall intracellular ROS. Since complexes I and III are the main contributors to mitochondrial ROS generation, it is not surprising that cancer cells carrying larger numbers of functional variations located in these two complexes would produce higher levels of mitochondrial ROS, as shown in this study.

Intriguingly, the positive correlation between the number of predicted complex I/III functional variations and drug IC50 seems more marked for cisplatin than for dequalinium. This suggests that cisplatin, a conventional nDNA-targeting compound, may rely more on elevated mitochondrial ROS to promote drug resistance in cancer cells compared to dequalinium, a mitochondria-targeting compound. Our observation was echoed by a recent study in which cisplatin-resistant lung cancer cells demonstrated increased mitochondrial mass through upregulation of PGC-1α (the predominant mitochondrial biogenesis promoter) and consequently enhanced intracellular ROS production upon cisplatin treatment [50]. Our data support the previously proposed theory that cancer cells may develop resistance towards cisplatin by upregulating mitochondrial ROS production in order to stimulate anti-apoptotic pathways and promote cell survival [50]. We speculate that this mechanism could be triggered by certain nucleus-mitochondria crosstalk in response to cisplatin treatment. On the contrary, unlike cisplatin, dequalinium is a mitochondria-targeting compound and therefore may not initiate such crosstalk, at least not to the same degree, upon entering the cells. This may explain the much lower IC50s of dequalinium observed in the cancer cells compared to the IC50s of cisplatin in the present study. Furthermore, our data and the previously published data support our hypothesis that mitochondria-targeting therapy can be more effective than conventional therapy since the latter may trigger nucleus-mitochondria crosstalk to promote cell survival.

Using 3D structural modelling, we predicted 8 functional OXPHOS variations: A10398G, T11120C, C12084T, A13681G, G13708A, C13802T, and A13966G are likely to destabilise complex I, impacting on corresponding enzyme activity, which in turn may affect ROS levels and drug response. In support of this view, A10398G was reported in various types of cancer including brain, breast and cervical cancer, and strongly associated with elevated levels of ROS [51–54]; T11120C and C13802T was found in the PC3 cells (but not the PNT-2 cells) previously and shown to inhibit OXPHOS and increase ROS production, and consequently promote tumour growth in vivo when those PC-3 cells were injected into nude mice [55]; C12084T and A13966G variations found in the MDA-MB-231 cells were previously reported to be responsible for reduced complex I activity and enhanced metastatic potential via ROS overproduction and the resultant overexpression of *Hif-1α* [56, 57]. While G13708A in complex I has been reported in various types of cancer such as breast and colorectal cancer [58, 59], A13681G has no previously reported disease associations. There are no previous reports documenting G13708A or A13681G and ROS levels, although our predictions suggest this would be worth investigating. On the other hand, we predict that the final functional OXPHOS mutation: T14798C, despite frequently occurring in the healthy population (like some of the other variations; Table S3), is likely to affect the association/dissociation of ubiquinone at the Qi-site, impacting on the activity of complex III, and this in turn may affect the levels of ROS produced by the complex and consequently drug response. In support of this view, a study conducted by Keatley et al. demonstrated that glioblastoma cell lines that carried T14798C had elevated complex III activity, oxidative stress, and different drug sensitivity levels compared to cells that did not contain the mutation. Further, glioblastoma patients with T14798C had worse prognosis than non-carriers [10]. This is in keeping with an earlier study which demonstrated that the activity of yeast mutant complex III genetically manipulated to contain the equivalent variation of T14798C was significantly impaired and the mutant showed more sensitivity to mitochondrial-targeting compounds [60].

In contrast to increased ROS and drug resistance levels correlating positively with greater mtDNAcn, larger number of non-synonymous complex I and III variations, and larger number of predicted complex I and III functional variations, none of these parameters correlated with the number of D-loop variations. The mtDNAcn result is surprising given that the D-loop region is a major control site for mtDNA replication and transcription, and higher mtDNAcn has been reported in various types of cancer carrying D-loop variations [61, 62]. One possibility is that specific variations located within the light and heavy strand promoters in the D-loop region rather than the total number of D-loop variations are more important. Such variations could affect the binding affinities of the initiators and modulators of mtDNA transcription, thus, disturbing the rate of transcription, RNA primer synthesis as well as mtDNA replication at the H-strand origin of replication (O_H_) [63]. In other words, quality is more important than quantity in terms of functional variations in the D-loop region. In the present study, only one variation appeared to be located in a key regulatory region. Indeed, 310InsC, present in Ishikawa, MDA-MB-231 and PC-3 cells, is located in CSB2, one of the three conserved sequence blocks within the H-strand origin of replication (O_H_) that harbours critical functional motifs for the initiation and regulation of H-strand replication. Given that insertion variations have a real fundamental impact on DNA function, as they completely change the sequence of the region and result in the shift of the original functional motifs, it is likely that the 310InsC variation may interfere with DNA-primer interaction and have a negative impact on the replication of the H-strand in those cell lines. This observation might explain why the above cell lines had lower mtDNA copy numbers compared to Caco-2 that did not harbour this insertion.

**Fig. 8:**
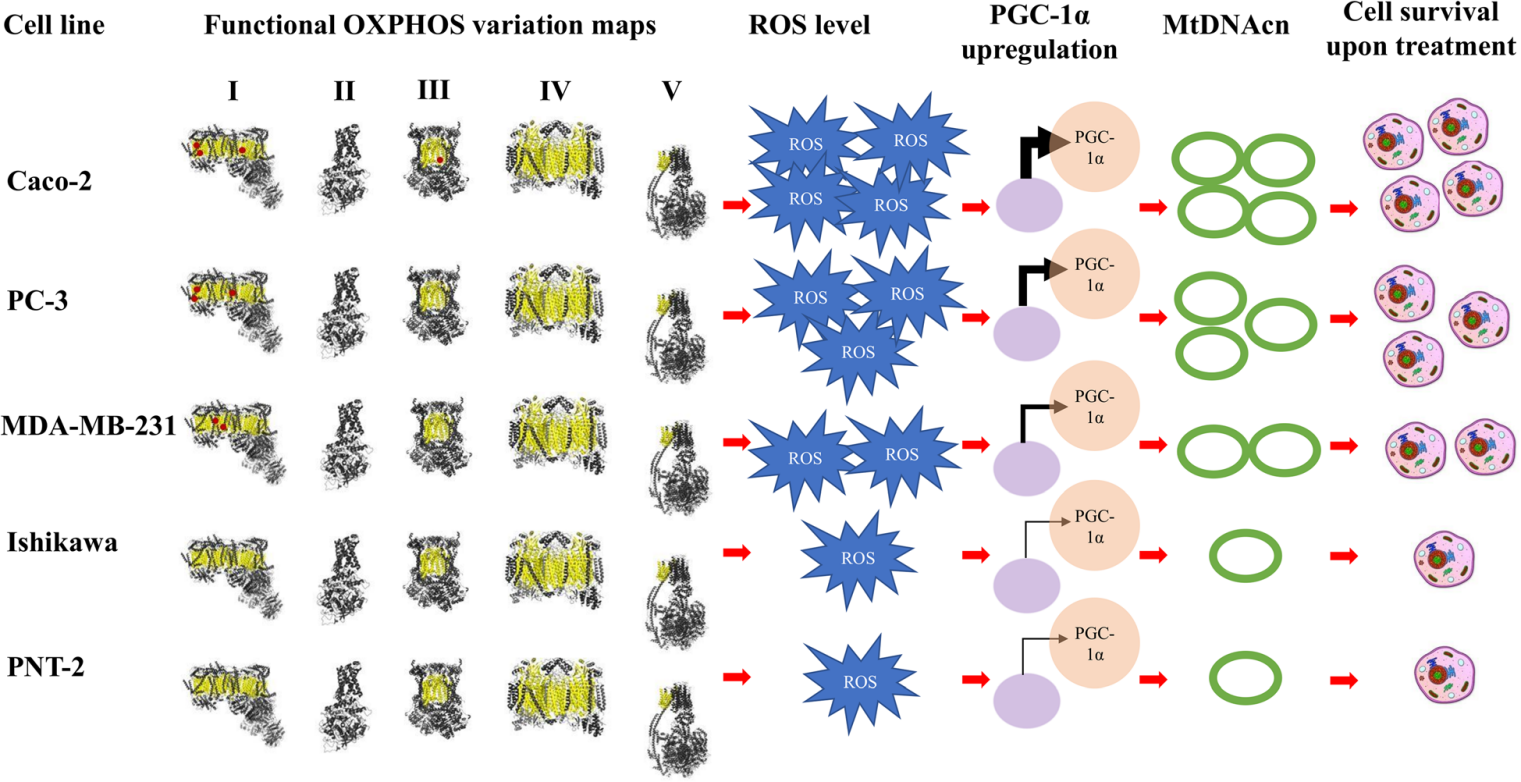
Proposed mechanism of functional OXPHOS mutation-mediated cell survival upon treatment with ROS-stimulating agents in cancer cells. The 3D-structures of OXPHOS complexes I-V with the nuclear-encoded subunits shown as ribbons (grey) and mitochondrial-encoded subunits shown as space filling model (yellow). Functional OXPHOS variations (red spheres) lead to the overproduction of ROS (blue) and mitochondrial dysfunction which in turn trigger cellular adaptation mechanisms by activating nuclear responses. In response to oxidative stress, nucleus (light purple) upregulates the transcription of genes responsible of mitochondrial biogenesis (e.g. PGC-1α, light orange) and mtDNA (open green circles) replication and transcription in order to compensate the mutated copies of mtDNA and avoid the over accumulation of ROS. The positive correlations between the total number of functional OXPHOS variations, ROS level, mtDNAcn and drug resistance observed in the present study indicate that oxidative stress could promote cancer cell survival via nucleus-mitochondria crosstalk

The apparent relationship between mtDNAcn, ROS levels and drug IC50s could also represent cellular adaptation to the level of OXPHOS activity that in turn might be related to the number of functional OXPHOS variations. One example of such adaptation includes the retrograde signalling pathway, where dysfunctional mitochondria (caused by functional OXPHOS variations) can lead to differences in ROS levels, which in turn lead to the activation of various nuclear responses, consequently promoting multiple pathways that regulate energy homeostasis, oxidative stress, mitophagy, fission, fusion and other functions to facilitate cellular adaption strategies and hence regulate the transcription and translation of genes responsible for mitochondrial biogenesis (e.g. PGC-1α), mtDNA replication and OXPHOS functions [64]. Taking these observations together, we propose that cell line-specific functional OXPHOS variation profiles could mechanistically contribute to the mitochondrial activity, baseline ROS levels, mtDNAcn and response to ROS-stimulating agents observed in cancer (Fig. 8), and the best illustration of this from our study is the comparison between the cancerous PC-3 cells and non-cancerous PNT-2 cells, as they are both derived from prostate.

A recent publication by Cocetta and co-authors provides a sophisticated review on mitochondrial involvement in cisplatin resistance [65]. As pointed out by the authors, so far, existing knowledge of the retrograde signalling is still very limited. Based on our data and the findings from a previous study linking PGC-1α upregulation with increased mitochondrial mass in cisplatin-resistant lung cancer cells [50], we present a hypothesis illustrated below in Fig. 8. Our data clearly indicate that specific functional variations in the OXPHOS complexes I and III are responsible for enhanced ROS production and the subsequent retrograde communication, rendering resistance to cisplatin. Our data not only support previous findings that redox imbalance and nucleus-mitochondria crosstalk play important roles in cisplatin resistance, but also provide a potential mechanistic link to specific mtDNA variations. Furthermore, our studies were not restricted to cisplatin only, hence our findings would provide insights into the broader area of ROS-stimulating/mitochondria-targeting therapy as well as the related biomarker studies.

Measuring mitochondrial activity and the expression level of genes controlling the mitochondrial biogenesis and mtDNA replication, such as PGC-1α, DNA polymerase γ (POLγ) and mitochondrial transcriptional factor A (TFAM), in the future would obviously help corroborate this hypothesis.

Now that we know the characteristics of the cell lines in detail, there are a number of approaches that could be employed in the future to further validate our hypothesis that functional OXPHOS variation or a group of variations contribute to the intracellular ROS/drug response phenotypes observed. One way would be to create cybrids which involves the transfer of the cancer cell mitochondria/mtDNA containing the functional OXPHOS variations onto a common *wild type* nuclear background, and the observation whether the cellular phenotype is transferred. However, the process of generating cybrids is not without its problems and can create irreversible epigenetic changes [66], as well as nuclear genome instability and variation [67], potentially confounding the ability to identify one single mtDNA variation (or a group of mtDNA variations) as the contributing factor(s), and so the insights generated in this paper, especially the comparison between PC-3 and PNT-2, are still useful. Another possibility would be to introduce the functional OXPHOS variations into *Saccharomyces cerevisiae* (Baker’s yeast) cells, as unlike mammalian mtDNA, its mitochondrial genome is amenable to genetic manipulation. This has proved useful for the functional validation of T14798C [60], but the lack of respiratory complex I in *S. cerevisiae* precludes a similar validation of functional complex I variations identified in this study. Recent technological advances suggest that such precise manipulation of mammalian mtDNA (and thus the functional validation of complex I variations) may be possible in the future, and may in fact provide a better alternative for functionally validating mtDNA variations than both cybrids and the yeast model system [68].

It is worth mentioning that two cell lines of distinctly opposite characteristics were identified among the tested cancer cell lines in our studies that was either the most sensitive (i.e. Ishikawa) or the most resistant (i.e. Caco-2) to ROS-stimulating therapy. The profiles of Ishikawa and Caco-2 based on the ROS/mtDNAcn/functional complexes I/III mutation parameters could be further validated as benchmarks to assess cancer cells’ response towards ROS-stimulating therapy. In addition, these two cell lines would make for interesting candidates for future downstream cybrid/mtDNA gene editing studies to help validate that the differences observed in the present study are due to mtDNA pattern and not due to differences in their nuclear proteomes.

## Conclusions

Our data showed significant correlations between mitochondrial genetic abnormalities and the baseline ROS levels and drug resistance in cancer cells. In particular, we highlight an association between elevated mtDNAcn, higher frequency of predicted functional non-synonymous variations in complexes I/III, elevated baseline ROS levels and enhanced drug resistance (Table S4). Consequently, screening patients for mtDNAcn and the key functional variations identified in this study (A10398G, T11120C, C12084T, A13681G, G13708A, C13802T, A13966G and T14798C) could provide healthcare professionals with useful information in terms of predicting a patient’s response to the treatment and designing personalised therapy in the future. It is worth mentioning that since the mtDNA mutations were identified using the Sanger sequencing method, we were unable to ascertain the level of heteroplasmy of these key functional variations. However, the fact that those variations were successfully detected by Sanger sequencing indicates that they are significantly abundant in the collective mitochondrial genome. Nevertheless, a more advanced technique such as Next Generation Sequencing would be able to provide such information in future studies and allow us to evaluate whether the extent of heteroplasmy also affects intracellular ROS and the associated drug sensitivity. Furthermore, the present study was carried out on a small number of samples, and we were unable to test more cell lines (particularly more paired cancer/non-cancerous cell lines) due to limited resources. However, our existing data clearly indicate the potential of the aforementioned mtDNA variations as biomarkers to predict cancer cells’ response to ROS-stimulating agents, and therefore warrant further investigations in this direction.

## Supplementary Information

**Additional file 1: Figure S1.** Representative image of agarose gel electrophoresis of PCR products. **Figure S2.** Correlation between the D-loop variations and the baseline intracellular ROS level, mitochondrial superoxide level, drug resistance against CDDP and DQA, relative content of mtDNA and the total number of variations. **Figure S3.** Correlation between the total variations and the baseline intracellular ROS level, mitochondrial superoxide level, drug resistance against CDDP and DQA and relative content of mtDNA. **Table S1.** List of common variations identified in the 5 cell lines. **Table S2.** List of unique variations identified in the 5 cell lines. **Table S3.** Summary of frequency in healthy tissues and heritability of functional variations identified in the 5 cell lines. **Table S4.** Summary of correlations between mtDNA parameters and ROS-stimulating drug IC50s, intracellular and mitochondrial ROS. **Table S5.** List of the primers designed to amplify 17 overlapping mtDNA fragments.

## Data Availability

Data generated or analysed during this study are included in this article and its supplementary information document. Sequencing data have been deposited to the NIH genetic sequence database GenBank and are accessible via the following accession numbers: MW344639 (PNT-2), MW344640 (Ishikawa), MW344641 (MD-MBA-231), MW344642 (PC-3), MW344643 (Caco-2). Other raw data used and/or analysed during the current study are available from the corresponding author upon reasonable request.

## Abbreviations

MRC: Mitochondrial respiratory chain
mtDNA: Mitochondrial DNA
OXPHOS: Oxidative phosphorylation system
nDNA: Nuclear DNA
D-loop: Displacement loop
TFAM: Mitochondrial transcriptional factor A
mtDNAcn: Mitochondrial copy number
SDH: Succinate dehydrogenase
ROS: Reactive oxygen species
CDDP: Cisplatin
DQA: Dequalinium chloride hydrate
HmtDB: Human Mitochondrial DataBase
RCSB PDB: Research Collaboratory for Structural Bioinformatics Protein Data Bank
COOT: Crystallographic Object-Oriented Toolkit
Qi site: Ubiquinone-binding site
F: Phenylalanine
L: Leucine
TM: Transmembrane
POLγ: DNA polymerase γ
