# Correction to: Mitochondrial DNA abnormalities provide mechanistic insight and predict reactive oxygen species-stimulating drug efficacy

**DOI:** 10.1186/s12885-021-08279-5

**Published:** 2021-05-10

**Authors:** Tarek Zaidieh, James R. Smith, Karen E. Ball, Qian An

**Affiliations:** 1 School of Pharmacy and Biomedical Sciences, Institute of Biological and Biomedical Sciences, University of Portsmouth, St Michael’s Building, White Swan Road, Portsmouth, PO1 2DT UK; 2 Institute of Life Science, Swansea University Medical School, Swansea, SA2 8PP UK

**Correction to: BMC Cancer 21, 427 (2021)**

https://doi.org/10.1186/s12885-021-08155-2

Following publication of the original article [1], the authors identified an error in the presentation of Fig. 4. The MT-ND1 subunit residue N382 is incorrect and should be D283. The correct Fig. 4 is supplied below.

**Fig. 4:**
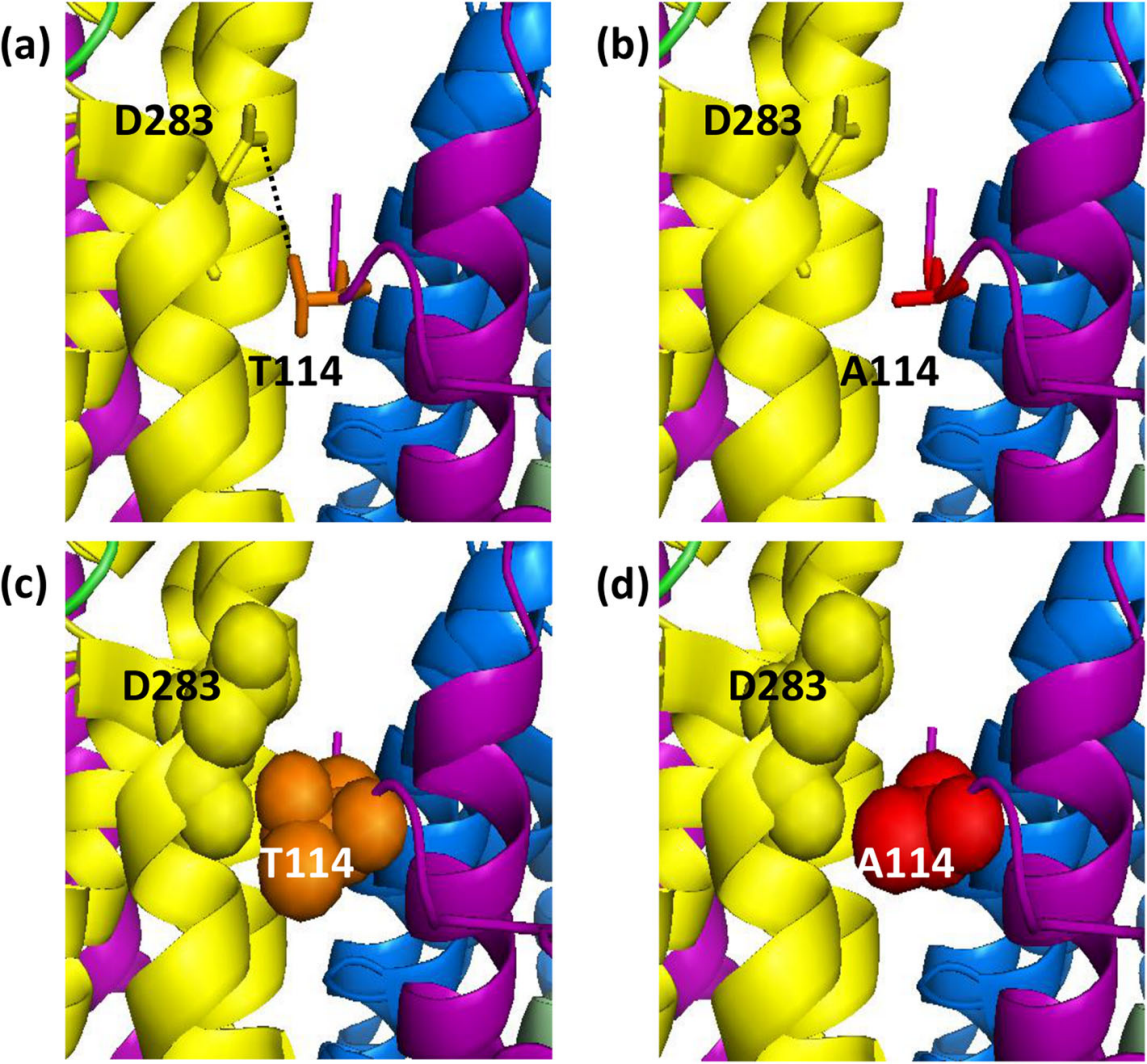
Detailed view of the complex I variation A10398G (T114A). T114 is located at the surface of complex I within the mitochondrial DNA encoded MT-ND3 subunit. MT-ND3 is shown in purple and MT-ND1, an adjacent subunit, in yellow. The *wild type* T114 is shown in orange as sticks and spheres (**a** & **c**, respectively) and the mutant A114 is shown in red (**b** & **d**). Alanine is non-polar and smaller than threonine in size meaning the change is likely to result in the loss of hydrogen bonds (dotted line) with the D283 residue of MT-ND1, and therefore affect the association of the two subunits and consequently the stability of complex I

The original article [1] has been corrected.
