# Supplementary material for: Mitochondrial DNA abnormalities provide mechanistic insight and predict reactive oxygen species-stimulating drug efficacy
Source: BMC Cancer. 2021 Apr 17;21:427. doi: 10.1186/s12885-021-08155-2 (PMC8053302; doi:10.1186/s12885-021-08155-2)
Supplement: Supplementary file 1 — Additional file 1: Figure S1. Representative image of agarose gel electrophoresis of PCR products. Figure S2. Correlation between the D-loop variations and the baseline intracellular ROS level, mitochondrial superoxide level, drug resistance against CDDP and DQA, relative content of mtDNA and the total number of variations. Figure S3. Correlation between the total variations and the baseline intracellular ROS level, mitochondrial superoxide level, drug resistance against CDDP and DQA and relative content of mtDNA. Table S1. List of common variations identified in the 5 cell lines. Table S2. List of unique variations identified in the 5 cell lines. Table S3. Summary of frequency in healthy tissues and heritability of functional variations identified in the 5 cell lines. Table S4. Summary of correlations between mtDNA parameters and ROS-stimulating drug IC50s, intracellular and mitochondrial ROS. Table S5. List of the primers designed to amplify 17 overlapping mtDNA fragments. [file 12885_2021_8155_MOESM1_ESM.pdf]

Mitochondrial DNA abnormalities provide mechanistic insight and predict reactive oxygen species-stimulating drug efficacy

Tarek Zaidieh[*], James R. Smith, Karen E. Ball, Qian An[*]

School of Pharmacy and Biomedical Sciences, Institute of Biological and Biomedical Sciences, University of Portsmouth, Portsmouth PO1 2DT, UK.
[*]Joint corresponding authors

Supplementary information

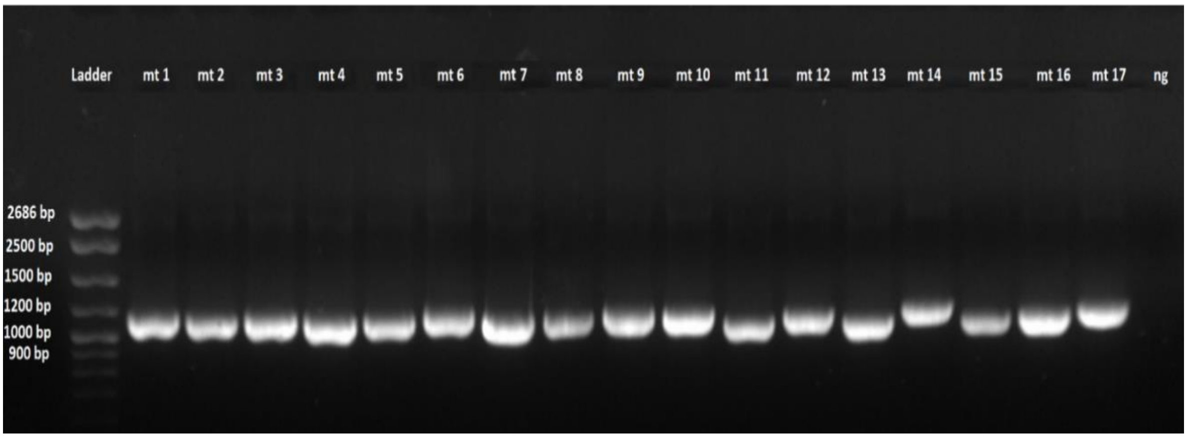

Figure S1. Representative image of agarose gel electrophoresis of PCR products amplified using 17 pairs of primers. This image was obtained from one single gel without any grouping or cropping. mt 1-17: seventeen fragments (~ 1,1 kb each) of mitochondrial genome; ng: negative control.

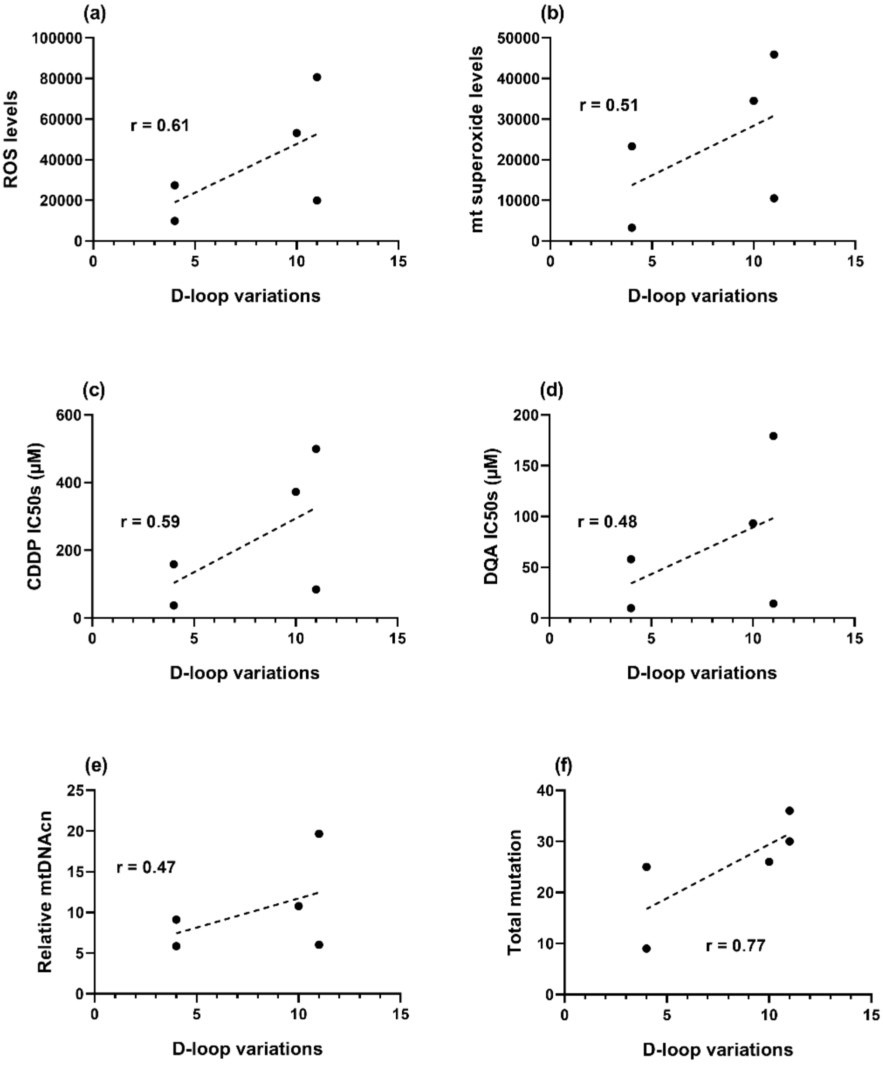

Figure S2. Correlation between the D-loop variations and the baseline intracellular ROS level (**a**), mitochondrial superoxide level (**b**), drug resistance against CDDP (**c**) and DQA (**d**), relative content of mtDNA (**e**) and the total number of variations (**f**). Data points representing the cell lines are in the sequence of PNT-2, Ishikawa, MDA-MB-231, PC-3 and Caco-2. r value was calculated using the Pearson correlation coefficient method.

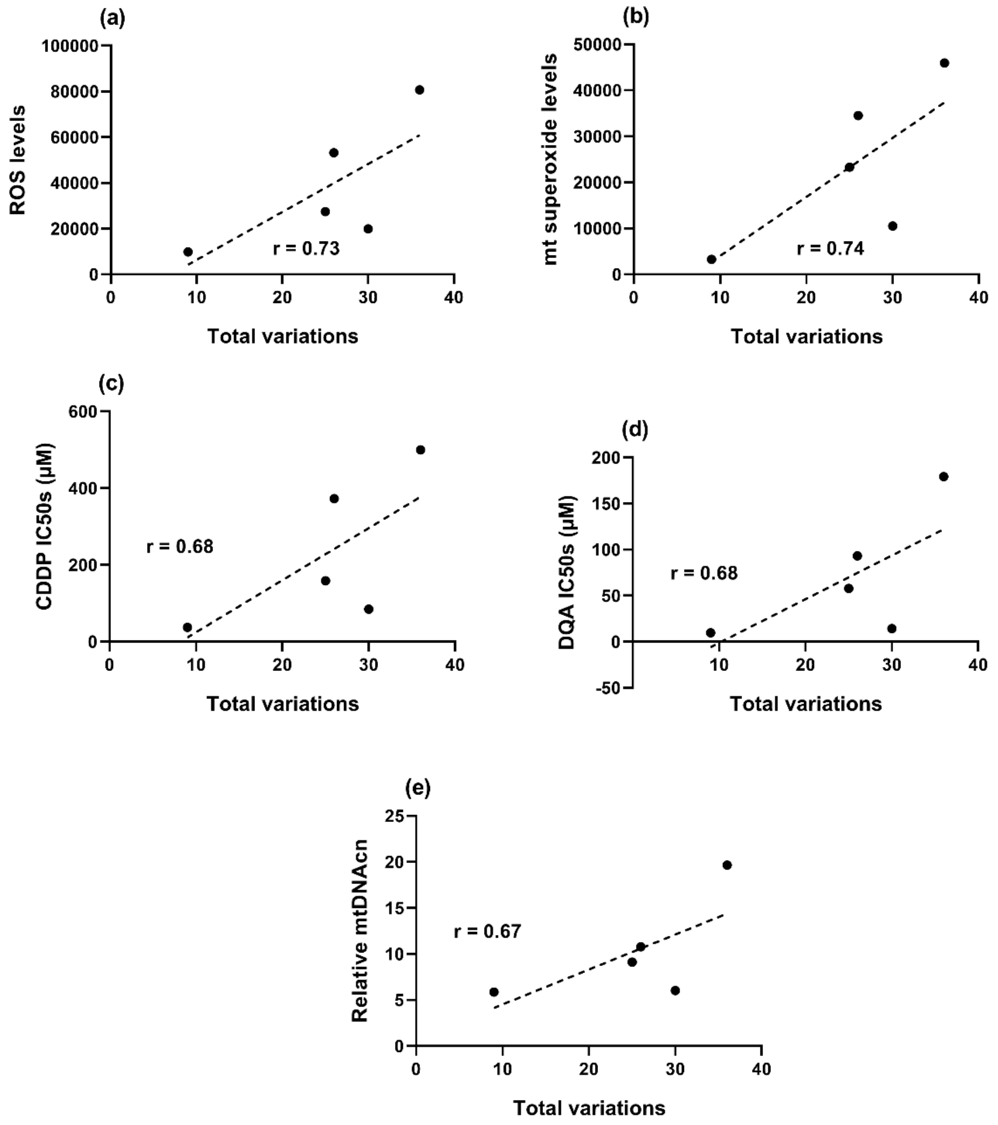

Figure S3. Correlation between the total variations and the baseline intracellular ROS level (**a**), mitochondrial superoxide level (**b**), drug resistance against CDDP (**c**) and DQA (**d**) and relative content of mtDNA. Data points representing the cell lines are in the sequence of PNT-2, Ishikawa, MDA-MB-231, PC-3 and Caco-2. r value was calculated using the Pearson correlation coefficient method.

Table S1. List of common variations identified in the 5 cell lines.

Variations	PNT-2	Ishikawa	MDA-MB-231	PC-3	Caco-2	Region	Variation type	Amino acid change
**A73G**		✓		✓	✓	D-loop	Non-protein coding	
**T195C**			✓	✓		D-loop	Non-protein coding	
**A263G**		✓	✓	✓	✓	D-loop	Non-protein coding	
**310InsC**		✓	✓	✓		D-loop	Non-protein coding	
**A750G**	✓	✓	✓	✓	✓	12S ribosomal RNA	Non-protein coding	
**A1438G**	✓	✓		✓	✓	12S ribosomal RNA	Non-protein coding	
**A2706G**		✓	✓	✓	✓	16S ribosomal RNA	Non-protein coding	
**2800InsA**		✓	✓			16S ribosomal RNA	Non-protein coding	
**3107delN**		✓	✓		✓	16S ribosomal RNA	Non-protein coding	
**A4769G**	✓	✓	✓	✓	✓	Complex I	Synonymous	
**C7028T**		✓	✓	✓	✓	Complex IV	Synonymous	
**A8860G**	✓	✓	✓	✓	✓	Complex V	Non-synonymous	Thr - Ala
**G11719A**		✓	✓	✓	✓	Complex I	Synonymous	
**G12372A**		✓		✓		Complex I	Synonymous	
**C12705T**		✓	✓			Complex I	Synonymous	
**G13708A**				✓	✓	Complex I	Non-synonymous	Ala -Thr
**C14766T**		✓	✓	✓	✓	Complex III	Non-synonymous	Thr -Ile
**A15326G**	✓	✓	✓		✓	Complex III	Non-synonymous	Thr -Ala
**T16172C**		✓		✓		D-loop	Non-protein coding	
**C16261T**		✓			✓	D-loop	Non-protein coding	

Table S2. List of unique variations identified in the 5 cell lines.

Variations	PNT-2	Ishikawa	MDA-MB-231	PC-3	Caco-2	Region	Variation type	Amino acid change
**513InsCA**	✓					D-loop	Non-protein coding	
**G16319A**	✓					D-loop	Non-protein coding	
**T16325C**	✓					D-loop	Non-protein coding	
**T16519C**	✓					D-loop	Non-protein coding	
**C150T**		✓				D-loop	Non-protein coding	
**C338T**		✓				D-loop	Non-protein coding	
**T961C**		✓				12S ribosomal RNA	Non-protein coding	
**A1937C**		✓				16S ribosomal RNA	Non-protein coding	
**T3394C**		✓				Complex I	Non-synonymous	Tyr - His
**G5231A**		✓				Complex I	Synonymous	
**G5417A**		✓				Complex I	Synonymous	
**T14968C**		✓				Complex III	Synonymous	
**C16223T**		✓				D-loop	Non-protein coding	
**C16257A**		✓				D-loop	Non-protein coding	
**T16304C**		✓				D-loop	Non-protein coding	
**A16497G**		✓				D-loop	Non-protein coding	
**A153G**			✓			D-loop	Non-protein coding	
**646InsA**			✓			tRNA-Phe	Non-protein coding	
**653InsT**			✓			12S ribosomal RNA	Non-protein coding	
**G709A**			✓			12S ribosomal RNA	Non-protein coding	

**Variations**	**PNT-2**	**Ishikawa**	**MDA-MB-231**	**PC-3**	**Caco-2**	**Region**	**Variation type**	**Amino acid change**
**G1719A**			✔			16S ribosomal RNA	Non-protein coding	
**T6221C**			✔			Complex IV	Synonymous	
**T8506C**			✔			Complex V	Synonymous	
**C12084T**			✔			Complex I	Non-synonymous	Ser - Phe
**A13966G**			✔			Complex I	Non-synonymous	Thr - Ala
**T14470C**			✔			Complex I	Synonymous	
**T15310C**			✔			Complex III	Synonymous	
**T11120C**				✔		Complex I	Non-synonymous	Phe - Leu
**A11467G**				✔		Complex I	Synonymous	
**A12308G**				✔		tRNA-Leu	Non-protein coding	
**T13617C**				✔		Complex I	Synonymous	
**C13802T**				✔		Complex I	Non-synonymous	Thr - Met
**A14793G**				✔		Complex III	Non-synonymous	His - Arg
**C16192T**				✔		D-loop	Non-protein coding	
**C16256T**				✔		D-loop	Non-protein coding	
**C16270T**				✔		D-loop	Non-protein coding	
**C16320T**				✔		D-loop	Non-protein coding	
**A16399G**				✔		D-loop	Non-protein coding	
**G47A**					✔	D-loop	Non-protein coding	

Variations	PNT-2	Ishikawa	MDA-MB-231	PC-3	Caco-2	Region	Variation type	Amino acid change
**G185A**					✔	D-loop	Non-protein coding	
**G228A**					✔	D-loop	Non-protein coding	
**C295T**					✔	D-loop	Non-protein coding	
**C462T**					✔	D-loop	Non-protein coding	
**T489C**					✔	D-loop	Non-protein coding	
**T4216C**					✔	Complex I	Non-synonymous	Tyr - His
**C6464A**					✔	Complex IV	Synonymous	
**C6554T**					✔	Complex IV	Synonymous	
**G7977C**					✔	Complex IV	Non-synonymous	Gly - Ala
**A10398G**					✔	Complex I	Non-synonymous	Thr - Ala
**A11251G**					✔	Complex I	Synonymous	
**G12127A**					✔	Complex I	Synonymous	
**A12612G**					✔	Complex I	Synonymous	
**A13681G**					✔	Complex I	Non-synonymous	Thr - Ala
**T14798C**					✔	Complex III	Non-synonymous	Phe - Leu
**A14927G**					✔	Complex III	Non-synonymous	Thr - Ala
**A14959G**					✔	Complex III	Synonymous	
**C15452A**					✔	Complex III	Non-synonymous	Leu - Ile
**C16069T**					✔	D-loop	Non-protein coding	
**T16126C**					✔	D-loop	Non-protein coding	

Table S3. Summary of frequency in healthy tissues and heritability of functional variations identified in the 5 cell lines.

Variations	3D Prediction	HmtDB*			mtDB*			MitoMap*		
		No. of complete genome entries	No. of entries with variation	Freq %	No. of complete genome entries	No. of entries with variation	Freq %	Freq %	Reported as somatic variation	Variants found in haplogroups at 50% or higher
T3394C	Non-functional	44058	475	1.08	2704	39	1.44	1.24	Yes	Yes
T4216C	Non-functional	44058	4334	9.84	2704	244	9.02	9.97	Yes	Yes
A10398G	Functional	44058	77	0.17	2704	1242	45.93	43.98	Yes	Yes
T11120C	Functional	44058	3	0.01	2704	0	0.00	0.00	No	No
C12084T	Functional	44058	48	0.11	2704	2	0.07	0.10	No	No
A13681G	Functional	44058	182	0.41	2704	10	0.37	0.39	No	No
G13708A	Functional	44058	3154	7.16	2704	174	6.43	7.20	Yes	Yes
C13802T	Functional	44058	34	0.08	2704	0	0.00	0.06	No	No
A13966G	Functional	44058	536	1.22	2704	31	1.15	1.26	No	Yes
C14766T	Non-functional	44058	n.i.a.[#]	n.i.a.	2704	2094	77.44	76.91	No	Yes
A14793G	Non-functional	44058	817	1.85	2704	39	1.44	2.00	No	Yes
T14798C	Functional	44058	2837	6.44	2704	218	8.06	6.56	Yes	Yes
A14927G	Non-functional	44058	146	0.33	2704	15	0.55	0.30	No	Yes
A15326G	Non-functional	44058	13363	30.33	2704	2687	99.37	98.67	No	Yes
C15452A	Non-functional	44058	4025	9.14	2704	235	8.69	9.33	No	Yes
G7977C	Non-functional	44058	6	0.01	2704	0	0.00	0.00	No	No
A8860G	Non-functional	44058	13567	30.79	2704	2698	99.78	98.48	No	Yes
310InsC	D-loop	44058	0	0.00	2704	n.i.a.	n.i.a.	0.00	No	No

*HmtDB, mtDB and MitoMAP were accessed on 15/10/2020.

[#]n.i.a.: No information available.

Table S4. Summary of correlations between mtDNA parameters and ROS-stimulating drug IC50s, intracellular and mitochondrial ROS. [#]

MtDNA parameter	CDDP IC50s	DQA IC50s	Intracellular ROS	Mitochondrial ROS
**mtDNAcn**	0.93*	0.99*	0.96**	0.92*
**CI/III ns variations**	0.94*	0.97**	0.97**	0.96*
**Functional CI/III ns variations**	0.96**	0.95*	0.94*	0.98**

[#] Values are expressed as correlation coefficients along with their relevant significance (*p*-values): *p<0.05 and **p<0.01. CI/III: complex I/III; ns: non-synonymous.

Table S5. List of the primers designed to amplify 17 overlapping mtDNA fragments.

Primer ID	Sequence	Length	Start Position	End Position	Size (bp)
F1	CCCTATTAACCACTCACGGG	20	17	36	1139
R1	CTGTGGCTCGTAGTGTTCTG	20	1155	1136	
F2	GATACCCCACTATGCTTAGCC	21	1079	1099	1114
R2	TTGGTGGCTGCTTTTAGGC	19	2192	2174	
F3	CTGGTGATAGCTGGTTGTCC	20	2006	2025	1131
R3	CTTGTCCTTTCGTACAGGGAG	21	3137	3117	
F4	GATCTGAGTTCAGACCGGAG	20	3063	3082	1103
R4	GTATGAGTTGGTCGTAGCGG	20	4165	4146	
F5	AACATATGACGCACTCTCCC	20	4041	4060	1108
R5	TCGTGGTGCTGGAGTTTAAG	20	5148	5129	
F6	ACCGTACAACCCTAACATAACC	22	5051	5072	1150
R6	GTTGTTTATGCGGGGAAACG	20	6200	6181	
F7	CCAGGCAACCTTCTAGGTAAC	21	6033	6053	1099
R7	CGTAGGTTTGGTCTAGGGTG	20	7131	7112	
F8	TGTAGCCCACTTCCACTATG	20	7022	7041	1109
R8	GTTTGGTTTAGACGTCCGGG	20	8130	8111	
F9	GTAGTACTCCCGATTGAAGCC	21	8009	8029	1153
R9	TAGGCTTGGATTAAGGCGAC	20	9161	9142	
F10	CATTACTGCAGGCCACCTAC	20	9015	9034	1136
R10	ATGTAGCCGTTGAGTTGTGG	20	10150	10131	
F11	ATAATCAACACCCTCCTAGCC	21	10080	10100	1070
R11	CAAGGTGGGGATAAGTGTGG	20	11149	11130	
F12	TCCAGTGAACCACTATCACG	20	11012	11031	1114
R12	CGGTAATGATGTCGGGGTTG	20	12125	12106	
F13	ACACCTATCCCCCATTCTCC	20	12076	12095	1072
R13	GGCTATTTTCTGCTAGGGGG	20	13147	13128	
F14	CAGCCCAATTAGGTCTCCAC	20	13007	13026	1168
R14	TAATTGAGATTGCTCGGGGG	20	14174	14155	
F15	TCCATCATCACCTCAACCCA	20	14056	14075	1081
R15	GCCTATGAAGGCTGTTGCTA	20	15136	15117	
F16	CAGAAACCTGAAACATCGGC	20	15075	15094	1112
R16	GGGTTTTGATGTGGATTGGG	20	16186	16167	
F17	GACTCACCCATCAACAACCG	20	16065	16084	1150
R17	TTTATGGGGTGATGTGAGCC	20	645	626	
